# The use of “brutacaine” in children in emergency departments in Denmark

**DOI:** 10.1186/1757-7241-20-S2-P49

**Published:** 2012-04-16

**Authors:** Michéle Lefort Sønderskov, Peter Hallas

**Affiliations:** 1 Anæstesiologisk Afd 4013, Juliane Marie Centret, Rigshospitalet, Denmark

## Background

The physical restraint of children during painful procedures has been called “brutacaine”. We sought to investigate if there was an un-met need for paediatric procedural pain management/sedation in Danish Emergency departments (EDs).

## Methods

An online questionnaire was distributed to the leading nurses of the Eds. We chose to limit our inclusion to the 21 emergency hospitals in Denmark.

## Results

The response rate was 81% (n=17). Of these, 71% (n=12) “often” or “sometimes” use physical restrain of children during procedures. Paediatric sedation is only possible in 29% of the EDs (n= 5). 29% (n=5) of the leading nurses stated that they at least once a month found there is a need for procedural sedation that their department is unable to provide. The three most commonly mentioned reasons for less than optimal pain management in the ER are: lack of subject specific education among the nursing staff of the EDs (71%, n=12) and lack of training among the doctors on call in the EDs on paediatric procedural sedation (53%, n=9) as well as a need for local guidelines on the subject (41%, n=7).

## Conclusion

Physical restrain of children is a common event in Danish ED’s. The findings indicate that there could be an un-met need for pain management during procedures in Danish EDs. Possible solutions could include guidelines on the subject, targeted education of ED staff and increased local involvement of the anaesthesia department concerning pain management in the ED’s.

